# RS-SCBiGRU: a noise-robust neural network for high-speed motor fault diagnosis with limited samples

**DOI:** 10.1038/s41598-025-02500-2

**Published:** 2025-07-02

**Authors:** Sun Fenghao, Li Guofa, He Jialong, Liu Shaoyang

**Affiliations:** 1 Key Laboratory of CNC Equipment Reliability, Ministry of Education, Jilin University, Changchun, 130025 China; 2 School of Mechanical and Aerospace Engineering, Jilin University, Changchun, 130025 China

**Keywords:** Fault diagnosis, Small sample, Data augmentation, Self-calibrating convolution, Bidirectional gated recurrent unit, Mechanical engineering, Mathematics and computing

## Abstract

Convolutional Neural Networks, with their excellent capabilities for automatic feature discrimination and learning, have been widely applied in the field of mechanical fault diagnosis. However, in real-world operating environments, acquiring large amounts of fault data as training samples is often challenging, which limits the applicability of traditional methods. To address this issue, this study proposes a frequency-adaptive fault diagnosis method for high-speed motors under small-sample scenarios. Specifically, this paper designs an innovative data augmentation technique that effectively expands the diversity and coverage of the training dataset and is seamlessly integrated into the fault diagnosis model. Furthermore, to enhance the richness of feature representations and strengthen information exchange between different feature channels, this paper proposes a frequency-adaptive convolutional layer (SCNET), which significantly optimizes the performance of Bidirectional Gated Recurrent Units (BiGRU) in fault feature extraction. Based on these technological improvements, we have constructed an efficient intelligent fault diagnosis model named RS-SCBiGRU. Experimental validation shows that, compared to various advanced fault diagnosis methods, the RS-SCBiGRU model achieves a significant improvement in accuracy and demonstrates stronger noise resistance capabilities.

## Introduction

The fault diagnosis of high-speed motor bearings holds significant engineering value in ensuring equipment safety, optimizing production efficiency, and extending service life. Accurate fault feature extraction and real-time condition monitoring can effectively suppress defect propagation, prevent secondary damage, significantly reduce maintenance costs, and ensure stable operation of production systems. By integrating intelligent diagnostic technologies into equipment maintenance strategies, a condition monitoring-based predictive maintenance system can be established, thereby substantially enhancing the reliability and operational efficiency of equipment management^1–5^.

With the advancement of technology, a large number of sensors are being employed for condition monitoring of mechanical equipment, and data-driven monitoring methods are receiving increasing attention from the industry. Traditional signal analysis methods are time-consuming and rely on professional expertise and manual analysis^6^. In contrast, intelligent fault diagnosis models have reduced their reliance on manual feature extraction. Methods such as convolutional neural networks^7^, autoencoders^8^, generative adversarial networks^9^, deep belief networks^10^, graph neural networks^11^, and recurrent neural networks^12^ have all demonstrated strong fault diagnosis capabilities. For example Guo et al.^13^, performed eigenvalue frequency domain calculations on speed signals from motor bearings and utilized convolutional neural networks to learn and classify the extracted features, achieving fault diagnosis of the bearings. Wan et al.^14^ designed a deep convolutional adversarial domain adaptation model that learns domain-invariant features through adversarial training, thereby improving the accuracy of fault diagnosis. Shao et al.^15^ and their team proposed an innovative improved convolutional deep belief network (CDBN) method based on compressed sensing (CS) technology, which was applied to the field of feature learning and fault diagnosis for rolling bearings. Yu et al.^16^ proposed a fault diagnosis framework based on graph neural networks (GNN) and a dynamic graph embedding mechanism (DGE), which addresses the issue of domain shift under varying operating conditions.

In addition, fault diagnosis under small-sample scenarios has become a research hotspot^17^. Shi et al.^18^ proposed a small-sample fault diagnosis method that integrates siamese networks with transfer learning. This method employs dual-input subnetworks to determine faults by comparing feature similarities and can leverage transfer learning for cross-operating-condition diagnosis. Fu et al.^19^ introduced a TWSCE-SSPN (a semi-supervised prototype network based on roller signals) that utilizes semi-supervised meta-learning to refine initial prototypes with unlabeled samples, thereby enhancing the classification accuracy of small-sample learning. Hu^20^ proposed a network for cross-domain small-sample fault diagnosis that employs hybrid attention to strengthen feature extraction and suppress redundancy. Qin et al.^21^ developed a siamese network with strong noise resistance capabilities, which can achieve fault diagnosis using a small number of sample data. Wu et al.^22^ presented an intelligent machine fault diagnosis method based on small-sample transfer learning, which, through meta-learning and transfer learning, can achieve accurate fault diagnosis with only a few fault samples. Li et al.^23^ utilized data augmentation techniques to expand the original fault data samples, addressing the issue of scarce fault samples and validating the effectiveness and superiority of their method in fault diagnosis. Wang et al.^24^ proposed a method for generating simulated fault data using generative adversarial networks (GANs), which, combined with deep neural networks for feature extraction and fault classification, can effectively identify different fault modes in planetary gearboxes, improving the accuracy and efficiency of fault diagnosis. Zhang et al.^25^ constructed simulation data based on dynamics to address the problem of insufficient training data. Additionally, while generated data can increase the number of training samples, this process involves complex computations, and the authenticity of the data remains to be investigated^26^.

Therefore, it is particularly important to fully leverage limited real data to generate more fault features. Currently, data augmentation methods can be employed to acquire more data features, such as the SMOTE oversampling technique^27^, noise addition^28^, and geometric transformations^29^. Gated RNN structures, with their excellent time-series modeling capabilities, not only can capture richer fault features but also effectively alleviate gradient vanishing or explosion issues, thereby facilitating effective learning and information transfer in models handling long sequence data^30^. Typical gated RNN structures include LSTM^31,32^ and GRU^33^. Compared to LSTM, GRU is widely favored due to its fewer parameters and lower computational costs. When dealing with fault diagnosis tasks that require consideration of backward dependencies, the bidirectional nature of BiGRU offers advantages over traditional GRU, which can only capture forward dependencies^34,35^. Self-calibrated convolution has been proven to effectively expand the receptive field and more accurately discriminate regions, improving fault diagnosis performance of rotating machinery in noisy environments^36,37^. In the field of fault diagnosis, combining self-calibrated convolution with other methods.

To achieve fault diagnosis with limited samples, this paper proposes a novel network model by integrating a resampling-based data augmentation strategy, frequency-adaptive convolution, and bidirectional gated recurrent units (BiGRU). The main contributions of this work are summarized as follows:

- *Fault Feature Enhancement Layer for Overfitting Mitigation* To address the overfitting issue caused by scarce training samples, a fault feature enhancement layer is proposed. This strategy enriches the diversity of input data features through multiple augmentation techniques, thereby improving the model’s generalization capability. Experimental results demonstrate that the proposed method effectively enhances fault diagnosis performance under small-sample conditions while alleviating overfitting.
- *Frequency-Adaptive Convolution for Variable-Length Data* A frequency-adaptive convolution method is introduced to enhance the scalability of vibration signal feature extraction. By capturing the intrinsic characteristics of vibration signals and effectively fusing cross-channel interaction information, this approach generates comprehensive feature representations. It not only enriches the connotation of fault features and improves feature description accuracy but also provides more robust and precise information for subsequent fault diagnosis.
- *RS-SCBiGRU: A Novel Fault Diagnosis Framework* By combining the data augmentation strategy, frequency-adaptive convolution, and BiGRU, a new fault diagnosis framework named RS-SCBiGRU is constructed. This framework enables more accurate fault feature extraction, leading to improved diagnostic precision and robustness.

The remainder of this paper is organized as follows: Sect. "Theoretical background" briefly introduces the fundamental theories of fault diagnosis. Section "The proposed model" elaborates on the overall workflow and architecture of the proposed method. Section "Verification and analysis" validates and discusses the performance of the proposed approach through two case studies. Finally, Sect. "Conclusion" concludes the paper and outlines future research directions.

## Theoretical background

### One-dimensional convolutional neural networks with large convolution kernels

One-Dimensional Convolutional Neural Networks with Large Convolution Kernels can extract richer fault features from raw signals and achieve noise filtering to a certain extent by utilizing large-sized convolution kernels. In the initial layer of the network, adopting large-sized convolution kernels helps the network capture both local and global structural information simultaneously^38,39^. During this process, convolution operations establish a specific correspondence between the input and output, which preserves spatial continuity and contextual information from the original signal. The correspondence between the input and output of convolution operations is as follows:

1 $$\begin{aligned} x_j^l = f\left( \sum _{i \in M_j} x_i^{l-1} * \alpha _{ij}^l + b_j^l\right) \end{aligned}$$

where $x_j^l$ represents the *j*-th feature map at the *l*-th layer, $f(\cdot )$ denotes the activation function, and $M_j$ indicates the set of feature maps. The pooling layer is positioned after the convolutional layer, serving to reduce data dimensionality and decrease computational complexity. It performs spatial downsampling on the output of the convolutional layer by applying pooling operations. This pooling operation helps extract dominant features while reducing the number of network parameters, thereby enhancing the model’s generalization capability. The pooling calculation formula is as follows:

2 $$\begin{aligned} x_{j}^{l} = \textrm{down}(x_{j}^{l - 1}) \end{aligned}$$

Here, l denotes the network layer where the data resides, j represents the position in the feature map, and down() indicates the downsampling operation. The activation function introduces nonlinearity into the network, enabling it to learn and model complex input-output mapping relationships. Commonly used activation functions include ReLU (Rectified Linear Unit), Sigmoid, and Tanh. These functions introduce nonlinearity to each neuron in the network, thereby enhancing its expressive power. By selecting appropriate activation functions, the network can better adapt to diverse and complex data distributions and patterns.

### Bidirectional gated recurrent unit

**Fig. 1:**
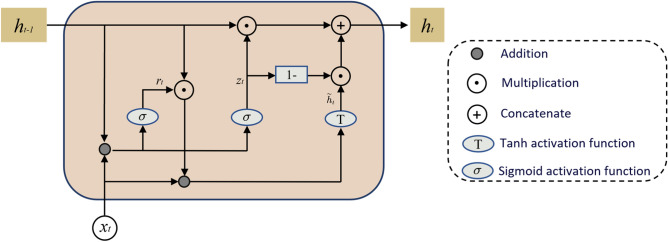
GRU structure.

The GRU architecture is compact with a relatively small number of parameters. As illustrated in Fig. 1.

the internal structure of a single GRU unit shows that for input data at time step t, the update rule for the GRU’s hidden state can be expressed as follows:

3 $$\begin{aligned} r_t = \sigma (W_r * [h_{t-1}, x_t]) \end{aligned}$$

4 $$\begin{aligned} z_t = \sigma (W_z * [h_{t-1}, x_t]) \end{aligned}$$

5 $$\begin{aligned} \tilde{h}_t = \tanh (W \cdot [r_t * h_{t-1}, x_t]) \end{aligned}$$

6 $$\begin{aligned} h_t = (1 - z_t) * h_{t-1} + z_t * \tilde{h}_t \end{aligned}$$

Where $\sigma$ denotes the sigmoid activation function, $r_t$ and $z_t$ represent the outputs of the reset gate and update gate at time *t*, respectively, $\tilde{h}_t$ is the candidate hidden state at time *t*, $W_r$, $W_z$, $W_h$ denote the weight matrices, $*$ indicates element-wise multiplication, and $[h_{t-1}, x_t]$ is the concatenation of the previous hidden state and current input.

Bidirectional GRU considers the context of the time series, and its uniqueness lies in its simultaneous consideration of both the forward and backward contextual information of the input sequence. Obviously, the formulas for the forward hidden state $\overrightarrow{h}_{t-1}$ and the backward hidden state $\overleftarrow{h}_{t-1}$ of the current hidden state at time t-1 are as follows:

7 $$\begin{aligned} & \overrightarrow{h}_t = G(x_t, \overrightarrow{h}_{t-1}) \end{aligned}$$

8 $$\begin{aligned} & \overleftarrow{h}_t = G(x_t, \overleftarrow{h}_{t-1}) \end{aligned}$$

9 $$\begin{aligned} & h_t = \left[ \overrightarrow{h}_t, \overleftarrow{h}_t \right] \end{aligned}$$

**Fig. 2:**
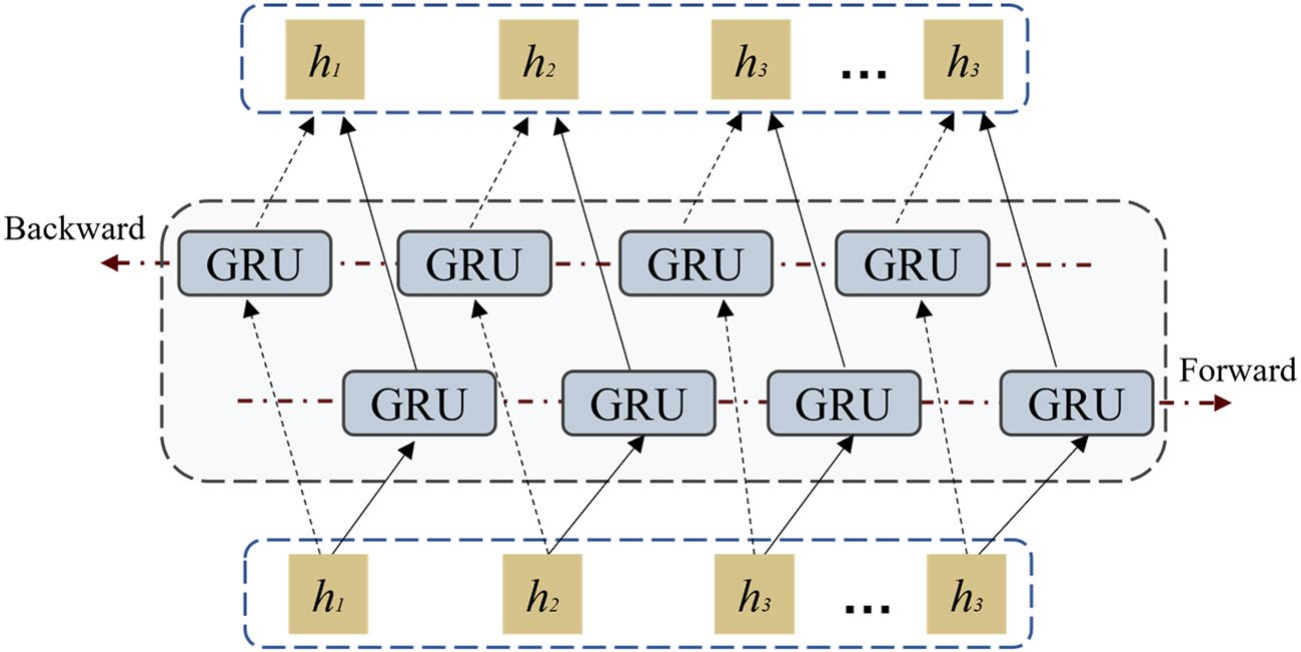
BiGRU calculation process.

Here, *G*() represents the nonlinear transformation operation within the GRU cell. Figure 2 illustrates the computational process of BiGRU.

This method increases data heterogeneity^40^ by introducing variations and perturbations into the original data. This approach helps to mitigate the overfitting phenomenon that occurs during the training of neural networks, where the model excessively fits the training data, leading to a decline in generalization performance on new data. By doing so, the model can enhance its predictive accuracy on unseen data while maintaining its ability to recognize features from the original data.

## The proposed model

### Feature enhancement layer

**Fig. 3:**
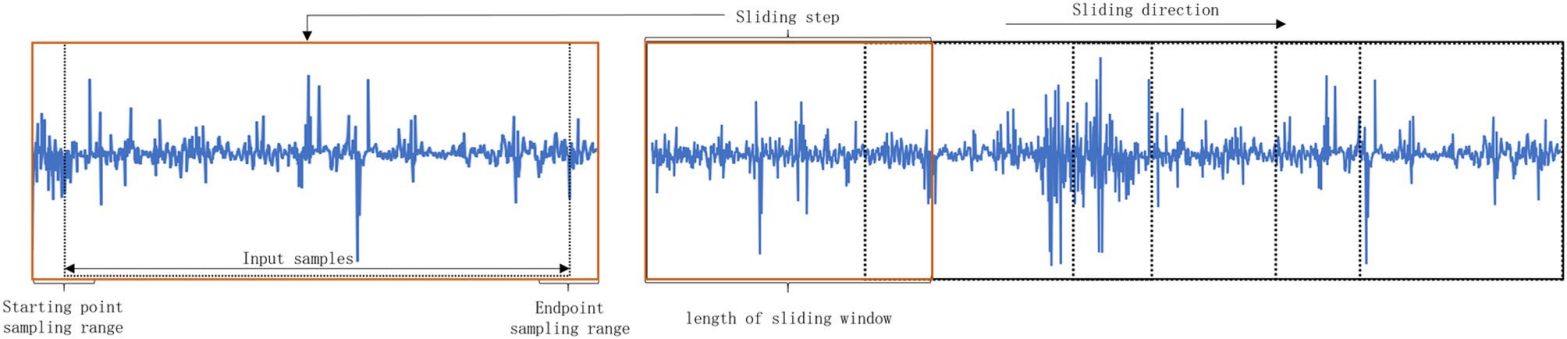
A schematic diagram of the random sampling process.

Inspired by resampling techniques and data augmentation methods, this paper proposes a Feature Enhancement Layer. This layer introduces a broader range of data diversity by randomly determining the length and starting point of a sequence to resample the input data into varying lengths. Subsequently, it employs one of three randomly selected data augmentation methods to enhance the resampled data. The random sampling process is illustrated in Fig. 3.

This layer is divided into the following two steps.

- Step 1: Random Sampling: In this step, the data undergoes preprocessing. The sample size $n = \left\lfloor \frac{N - l}{m} \right\rfloor + 1$ is calculated based on the input size *l*, the sliding step size *m*, and the signal length *N*. The data length $L_i = \lfloor q \cdot l \rfloor$ is randomly resampled according to a scaling factor q, and a starting point $X \sim \mathcal {U}(0, N - L_i)$ is randomly chosen to ensure data diversity.
- Step 2: During the model training phase, the input data randomly selects one of the following three processing methods with probability *p*: *GaussianNoiseInjection*: Gaussian white noise with a mean of zero and a variance of $\textbf{x}$ is added to the sampled data $\sigma ^2$ :
  10 $$\begin{aligned} \textbf{x}' = \textbf{x} + \epsilon , \quad \epsilon \sim \mathcal {N}(0, \sigma ^2) \end{aligned}$$
  where $\sigma$ is a control parameter for the noise intensity. *RandomScaling*: A random linear transformation is applied to the data amplitude, with the scaling factor $\sigma$ following a uniform distribution:
  11 $$\begin{aligned} \textbf{x}' = \alpha \textbf{x}, \quad \alpha \sim \mathcal {U}(\beta _{\text {min}}, \beta _{\text {max}}) \end{aligned}$$
  The default settings are $\beta _{\text {min}}=0.9$, $\beta _{\text {max}}=1.1$ to maintain the stability of the data distribution. $Original Data Pass-through$: With a probability of $1-p$, the unenhanced data is directly inputted to ensure that the model also learns from the original features.

### Frequency-adaptive convolution

**Fig. 4:**
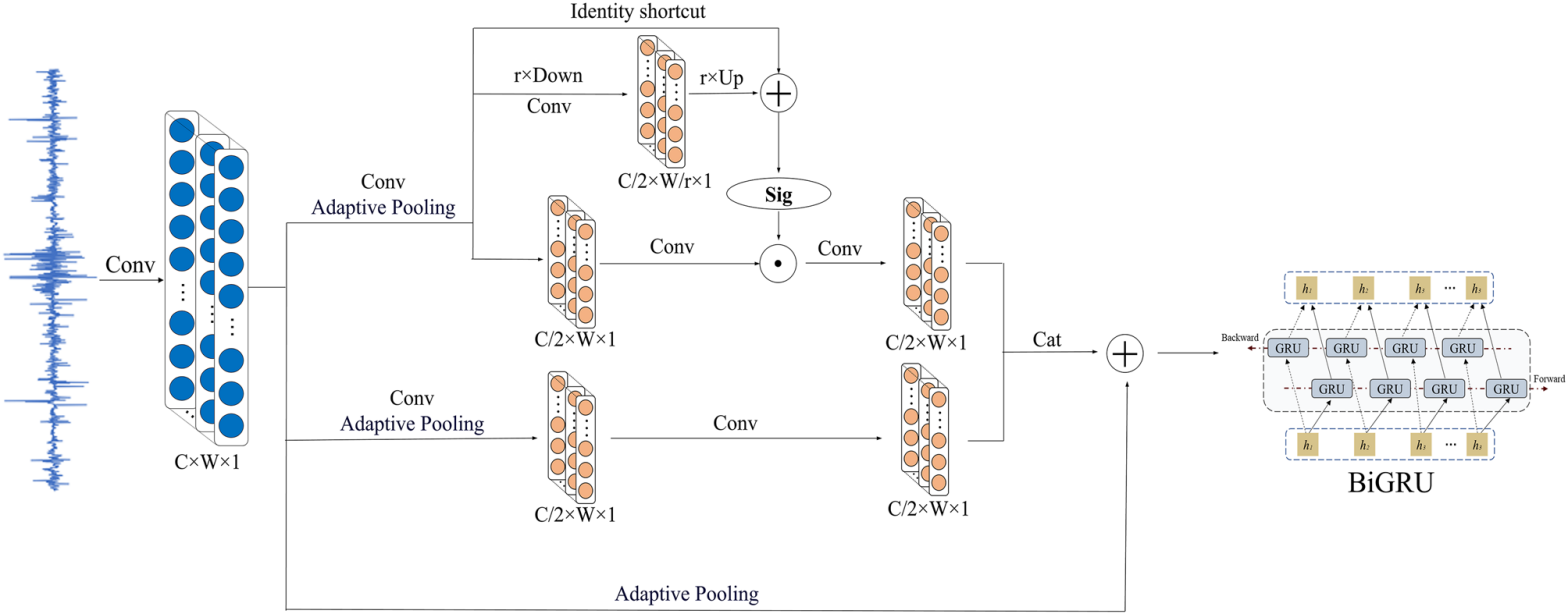
Schematic diagram of frequency adaptive convolution.

Given the length-invariant property of one-dimensional convolution operations, the network designed in this paper employs a one-dimensional large convolution kernel in the initial layer to process the input data. This design effectively mitigates the interference of noise on the model’s performance. Subsequently, the fault features are fed into the proposed frequency-adaptive convolution model, which has the capability to dynamically adjust the convolution kernel parameters, thereby adapting to data features of varying lengths. A schematic diagram of its operation is illustrated in Fig. 4.

The frequency-adaptive convolution achieves diversified extraction of feature frequencies through three distinct pathways. In the first pathway, convolution operations and maximum adaptive pooling are performed specifically for fault characteristics, where the number of feature channels after convolution is reduced to half of the original. Let *X* denote the features obtained from the initial data convolution; the frequency-adaptive process is expressed by the formula:

12 $$\begin{aligned} T_{1}= & \text {Conv}(X) \end{aligned}$$

13 $$\begin{aligned} T_{2}= & \text {AdaptiveMaxPool}(T_{1}) \end{aligned}$$

14 $$\begin{aligned} T_{3}= & \text {AvgPool}_r(T_{2}) \end{aligned}$$

15 $$\begin{aligned} T_{4}= & \text {Up}(\text {Conv}(T_{3})) + T_{2} \end{aligned}$$

16 $$\begin{aligned} C_{1}= & \sigma (T_{4}) + \text {Conv}(T_{2}) \end{aligned}$$

Among them, the fault features after convolution adaptive pooling are denoted as $T_2$, *r* represents the receptive field and stride of the pooling layer, *UP*() is a bilinear interpolation operator that maps the intermediate reference from a small-scale space to the original feature space, and $\gamma$ stands for the Sigmoid activation function. In the second path, two convolution operations and maximum adaptive pooling are applied to the fault features, with the number of feature channels being the same as in the first path. All channels from $C_1$ and $C_2$ are then concatenated, and the calculation process is described by the following formula:

17 $$\begin{aligned} C_{2}= & \text {Conv}(\text {AdaptiveMaxPool}(\text {Conv}(X)))\end{aligned}$$

18 $$\begin{aligned} Y= & \text {cat}(C_{1}, C_{2}) \end{aligned}$$

The third path integrates the fault features to ensure that their channel count and length are consistent with *Y*, and then superimposes the data from all three paths. The calculation process is described by the following formula:

19 $$\begin{aligned} \text {Out} = Y + \text {AdaptiveMaxPool}(X) \end{aligned}$$

By fusing the features from the three paths, information exchange between channels is promoted, introducing richer representation methods to the model. The regularization effect generated by this process will be systematically verified in subsequent experiments.

### Fault diagnosis procedure

**Fig. 5:**
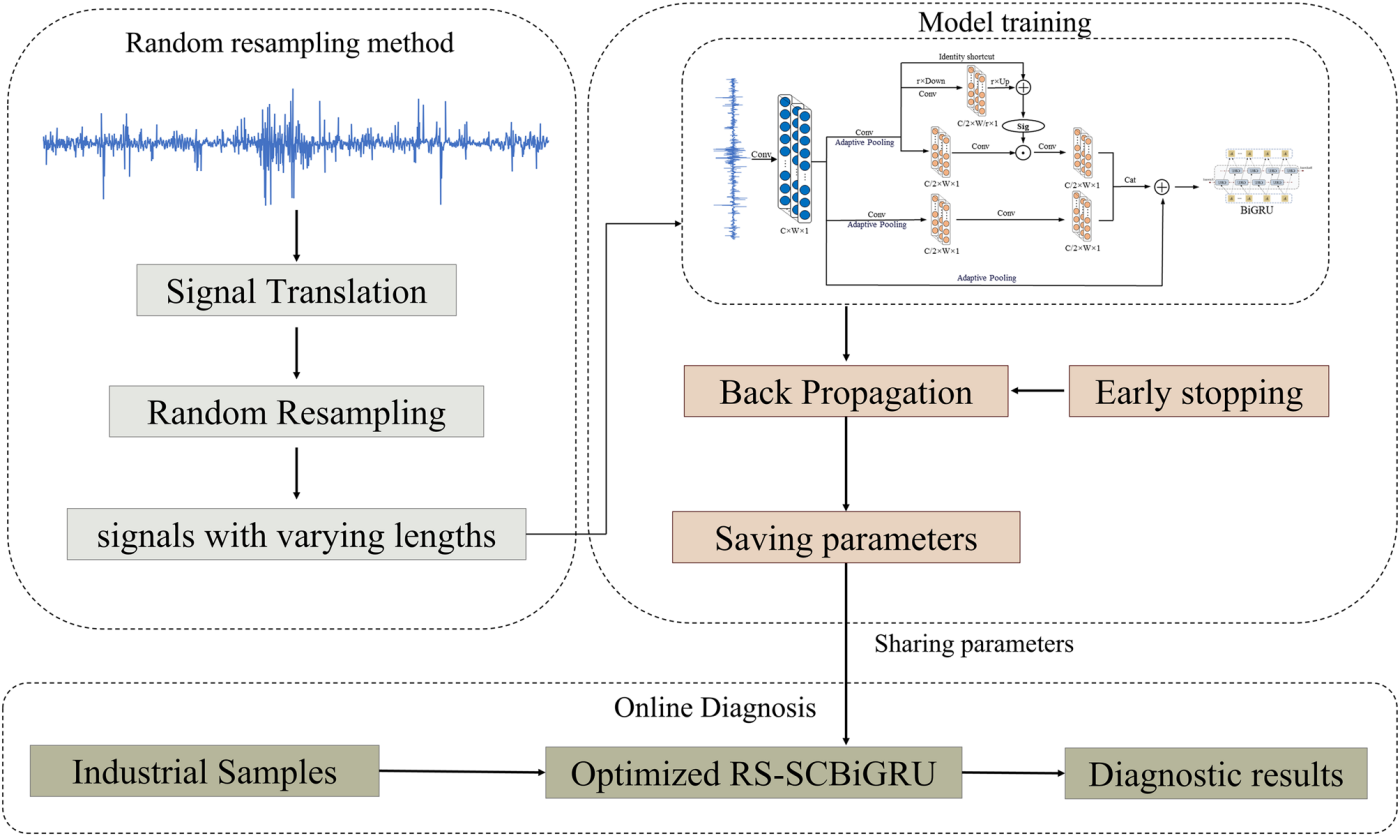
Proposed fault diagnosis framework.

In the field of fault diagnosis, the comprehensive utilization of multiple network optimization techniques combined with the intrinsic morphological and structural characteristics of input data has been demonstrated to effectively achieve accurate fault identification. Although the bidirectional gated recurrent unit (BiGRU) algorithm has been applied to some extent in this domain, its diagnostic accuracy and noise suppression capability when processing small-sample data still require further optimization. To address these challenges, this study proposes an intelligent fault diagnosis method named SC-BiGRU, which integrates random resampling, frequency-adaptive convolution, attention mechanisms, BiGRU, and global average pooling (GAP). The framework and components of this method are illustrated in Fig. 5.

The structural parameters of the model proposed in this paper are shown in Table 1:

Table 1: Model Structural Parameters

**Table 1:** Architecture of the proposed fault diagnosis framework.

Layer	Type	In channels	Out channels	Kernel size	Stride	Input size	Output size
1	Conv1d	1	16	64	8	2048	256
2	MaxPool1d	–	–	2	2	256	128
3	BatchNorm1d	16	–	–	–	128	128
4	SC Conv1d	16	16	–	–	128	64
5	Conv1d	16	32	3	1	64	64
6	MaxPool1d	–	–	2	2	64	32
7	BatchNorm1d	32	–	–	–	32	32
8	Conv1d	32	64	3	1	32	32
9	MaxPool1d	–	–	2	2	32	16
10	BatchNorm1d	64	–	–	–	16	16
11	Conv1d	64	64	3	1	16	16
12	MaxPool1d	–	–	2	2	16	8
13	BatchNorm1d	64	–	–	–	8	8
14	BiGRU	16	64	–	–	128	128
15	Linear	64	10	–	–	128	10
16	AdaptiveAvgPool1d	–	–	–	–	10	1
17	Conv1d	64	64	1	1	32	32

## Verification and analysis

The proposed intelligent fault diagnosis model was implemented using PyTorch 1.13.1 and Python 3.8.16. All computational experiments were conducted on a hardware system equipped with an AMD Ryzen 5 2600 Six-Core Processor (3.85 GHz) and an NVIDIA GeForce RTX 3060 Ti GPU to accelerate model training and inference.

### Evaluation methods for intelligent fault diagnosis models

The F1 Score is the harmonic mean of precision and recall, used to comprehensively evaluate the performance of a classifier. It ranges from 0 to 1, with higher values indicating better classifier performance.

### High-speed motor bearing data from Jilin University

**Fig. 6:**
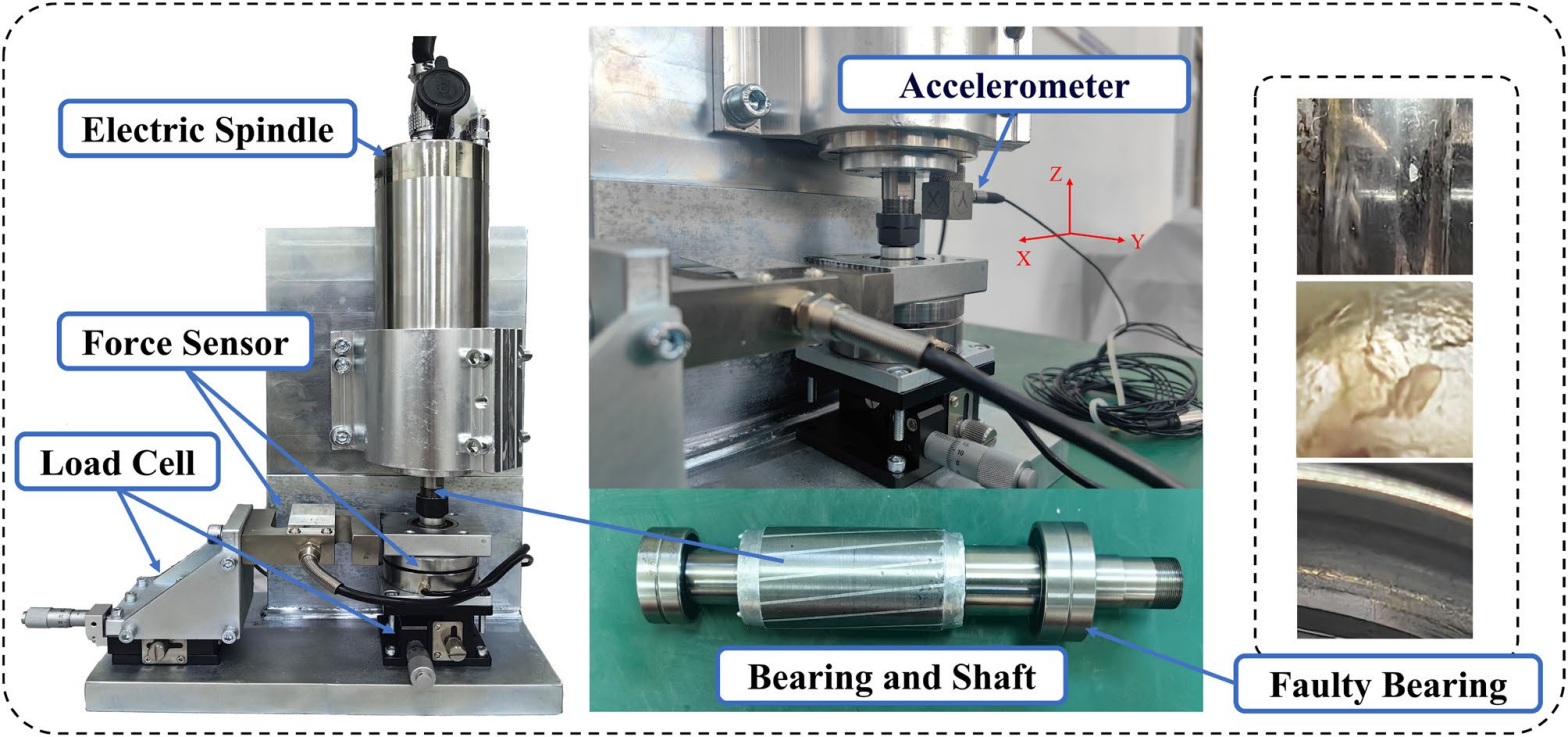
Jilin University high speed motor fault simulation experiment platform.

To validate the proposed methodology under realistic operating conditions, experimental investigations were conducted using high-speed electric spindle bearing data obtained from Jilin University. The fault patterns employed in this study were derived from actual industrial bearing failures, ensuring the representativeness of the experimental data. As illustrated in Fig. 6, the experimental setup features a compact electric spindle as its core component. The defective bearing was installed within the spindle assembly, where a precision sliding table system was employed to apply controlled axial and radial loads to the angular contact bearing. These mechanical loads were subsequently transmitted to the electric spindle through the angular contact bearing mechanism.

To verify the fault diagnosis accuracy of the model under small-sample conditions, the network was trained using 10 training samples, with the data composition shown in Table 2.

**Table 2:** Fault diagnosis dataset specifications.

Data type	Label	Dataset A load (N)	Dataset B load (N)	Dataset C load (N)
Normal	0	0	100	200
Inner race wear	1	0	100	200
Rolling element wear	2	0	100	200
Outer race wear	3	0	100	200
Inner race + Rolling element Wear	4	0	100	200
Inner race + Outer race wear	5	0	100	200
Rolling element + Outer race wear	6	0	100	200

**Fig. 7:**
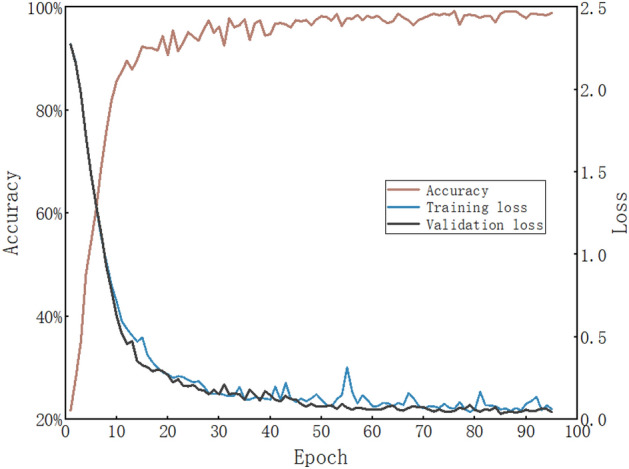
The relationship between iteration times, accuracy, and loss.

In model training, cross-entropy was employed as the loss function to optimize performance. To prevent overfitting, an Early-Stopping mechanism was introduced, with a maximum training iteration limit set at 100 and a patience value of 10. The Adam optimizer was utilized, with a small learning rate of 1e-3 to ensure stable training. For enhanced computational efficiency, the batch size was set to 10. Figure 7 illustrates the changes in loss and accuracy for both the training and validation sets, reflecting the model’s effective learning of data features and its good generalization performance.

#### Variable load experiment

In real-world industrial environments, fault data often encounter variable load conditions, making it essential to conduct variable load experiments on the model. Datasets A, B, and C represent the vibration data of bearings operating under loads of 0N, 100N, and 200N, respectively. To demonstrate the significant advantages of the proposed method in the field of small-sample fault diagnosis, this paper compares it with state-of-the-art intelligent diagnostic models. For fairness, all models utilize datasets from the same source and follow identical training parameters and methods. The comparative methods include Method 1 (BiGRU)^41^, Method 2 (BiLSTM)^42^, Method 3 (DAMN)^43^, Method 5 (MSCNN)^44^, Method 6 (MSCNN-LSTM)^45^, Method 7 (RNN-WDCNN)^46^, and Method 8 (WDCNN)^47^. To further validate the effectiveness of the random resampling method, this study introduces SCBiGRU as a comparative model. It is worth noting that, except for not adopting the random resampling strategy, all parameter configurations of SCBiGRU are consistent with those of the proposed method. This design ensures the fairness and accuracy of the experimental results, focusing solely on the impact of the random resampling strategy on model performance. To ensure the accuracy of the experimental results and mitigate the influence of randomness, the experiment was repeated ten times, and the average of these results was used as the final data. The experimental results are presented in Table 3.

**Table 3:** Performance comparison of different methods.

Methods	A→B		A→C		B→A		B→C	
	Accuracy (%)	F1 (%)	Accuracy (%)	F1 (%)	Accuracy (%)	F1 (%)	Accuracy (%)	F1 (%)
Proposed method	79.20	72.37	86.04	83.73	77.02	69.86	77.56	71.25
SCBiGRU	74.13	68.03	83.06	81.46	75.71	68.58	77.11	70.03
BiGRU	74.65	68.16	80.73	78.42	77.04	69.96	78.07	71.68
BiLSTM	75.82	69.72	82.22	79.28	76.31	69.47	76.58	69.51
DAMN	77.09	70.75	77.33	72.38	79.80	73.33	78.64	72.31
MSCNN	79.69	73.21	82.76	78.83	76.85	73.81	71.49	55.97
MSCNN-LSTM	62.31	58.25	64.36	60.02	64.33	57.69	62.40	55.90
RNN-WDCNN	72.91	66.77	82.73	80.86	72.69	66.14	71.67	65.87
WDCNN	62.73	57.51	74.58	72.07	62.47	54.78	62.78	55.66

In order to minimize the impact of randomness on experimental results and ensure their reliability, this study repeated the experiment 10 times and took the mean values of accuracy and F1 score as the final experimental results. This approach effectively enhanced the stability of the results.

**Fig. 8:**
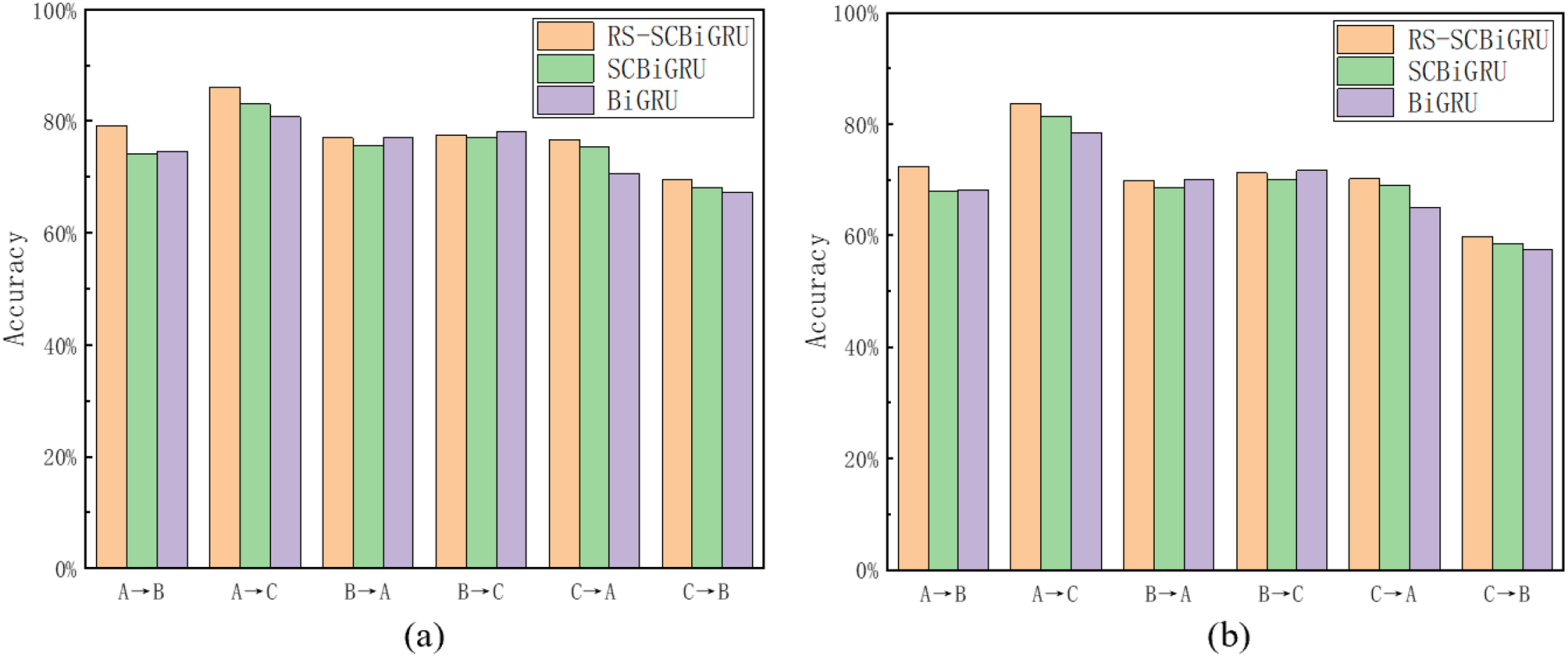
Variable load test results.

The comparison chart of experimental results is shown in Fig. 8. By comparing the method proposed in this paper with the method without random resampling, it can be observed that the accuracy and F1 score of the proposed model are significantly higher than those of the comparative method, demonstrating the effectiveness of the method proposed in this paper.

**Fig. 9:**
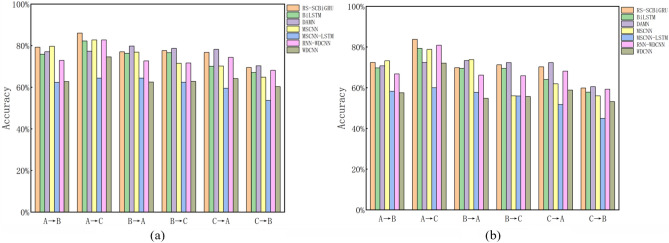
Comparison results with other advanced fault diagnosis methods.

The experimental results demonstrate that the average accuracy of the RS-SCBiGRU method for variable load prediction has increased by 2.11% compared to the SCBiGRU method and by an even more substantial 2.97% when compared to the traditional BiGRU method. Furthermore, when evaluating the overall performance of the model, the F1 score of the RS-SCBiGRU method surpasses that of the SCBiGRU method by 1.92% and outperforms the BiGRU method by 2.76%. A comparison with other methods is illustrated in Fig. 9:

An analysis of the comparative data reveals that the proposed method achieves a 3.00% improvement over BiLSTM, a 0.78% enhancement over DAMN, a 3.38% increase compared to MSCNN, a substantial 16.59% improvement over MSCNN-LSTM, and notable advancements of 3.93% and 13.18% over RNN-WDCNN and WDCNN, respectively. These results demonstrate the method’s superiority and practicality.

#### Analysis of noise robustness

To simulate data from real-world industrial scenarios, Gaussian white noise with varying signal-to-noise ratios (SNRs) is added to the test set data during the experiment. The specified SNRs are set to 0, 2, 4, 6, 8, and 10, and the calculation method for the SNR is as shown in Eq. (20).

20 $$\begin{aligned} \text {SNR} = 10 \lg \left( \frac{P_{S}}{P_{N}} \right) \end{aligned}$$

**Fig. 10:**
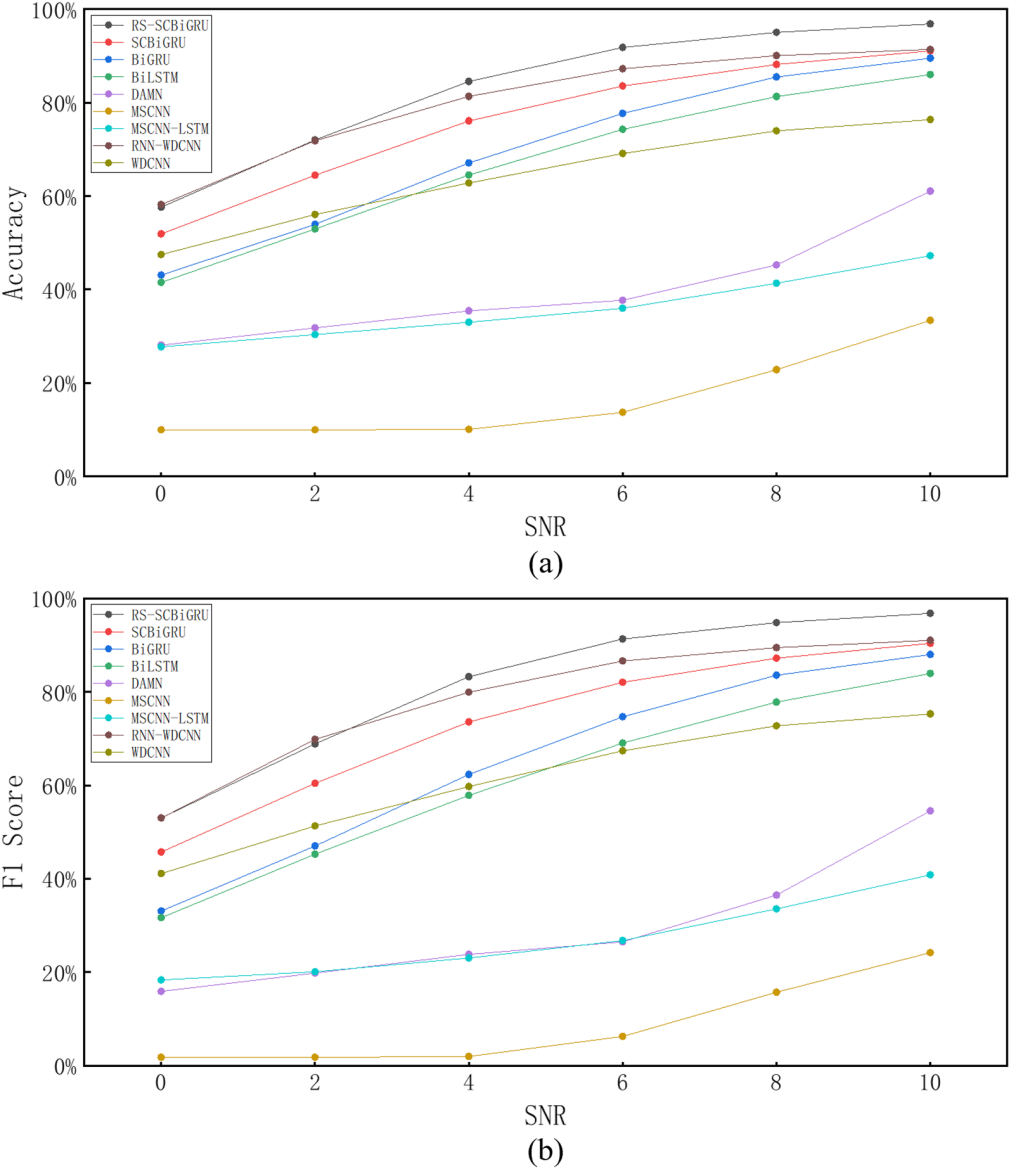
Test results of different signal-to-noise ratios.

Here, $P_S$ represents the energy of the vibration signal, and $P_N$ denotes the energy of the noise signal. The test data is generated by superimposing a series of noise data with varying signal-to-noise ratio (SNR) values onto the original test data. To comprehensively illustrate the noise resistance capability of the proposed method, test data with different SNRs are employed to evaluate various advanced fault diagnosis methods. The test results are presented in Table 4 and Fig. 10.

**Table 4:** Performance comparison of different methods under varying signal-to-noise ratios.

Methods	0		2		4		6		8	
	Accuracy (%)	F1 (%)	Accuracy (%)	F1 (%)	Accuracy (%)	F1 (%)	Accuracy (%)	F1 (%)	Accuracy (%)	F1 (%)
Proposed method	57.65	53	72.00	68.89	84.49	83.28	91.80	91.31	95.04	94.84
SCBiGRU	51.91	45.75	64.44	60.45	76.05	73.59	83.54	82.08	88.13	87.22
BiGRU	43.11	33.10	53.94	47.03	67.06	62.31	77.69	74.71	85.47	83.60
BiLSTM	41.56	31.66	52.98	45.29	64.51	57.84	74.27	69.06	81.27	77.83
DAMN	28.13	15.91	31.82	19.81	35.45	23.84	37.71	26.53	45.29	36.52
MSCNN	10.00	1.82	10.00	1.82	10.09	1.99	13.73	6.28	22.87	15.72
MSCNN-LSTM	27.76	18.35	30.36	20.11	33.02	23.04	36.00	26.80	41.35	33.57
RNN-WDCNN	58.18	53.01	71.84	69.86	81.31	79.95	87.25	86.62	90.04	89.49
WDCNN	47.51	41.11	56.04	51.30	62.78	59.76	69.09	67.41	73.95	72.77

Through testing and verification, it has been observed that traditional intelligent diagnostic methods exhibit significant deficiencies in noise resistance when there is a severe shortage of training samples, as they are unable to adequately learn the characteristics of vibration data. In contrast, the method proposed in this paper demonstrates a remarkable advantage in terms of accuracy. Specifically, when compared to methods such as SCBiGRU, BiGRU, BiLSTM, DAMN, MSCNN, MSCNN-LSTM, RNN-WDCNN, and WDCNN, the accuracy of the proposed method has improved by 7.11%, 13.51%, 16.20%, 43.06%, 66.28%, 47.01%, 2.97%, and 18.68%, respectively. Additionally, the proposed method also shows a clear advantage in the key evaluation metric F1 score, with improvements of 8.10%, 16.56%, 20.42%, 51.83%, 72.72%, 54.24%, 3.03%, and 20.09% compared to the aforementioned methods, respectively.

### High-speed motor bearing data from Politecnico di Torino^48^

**Fig. 11:**
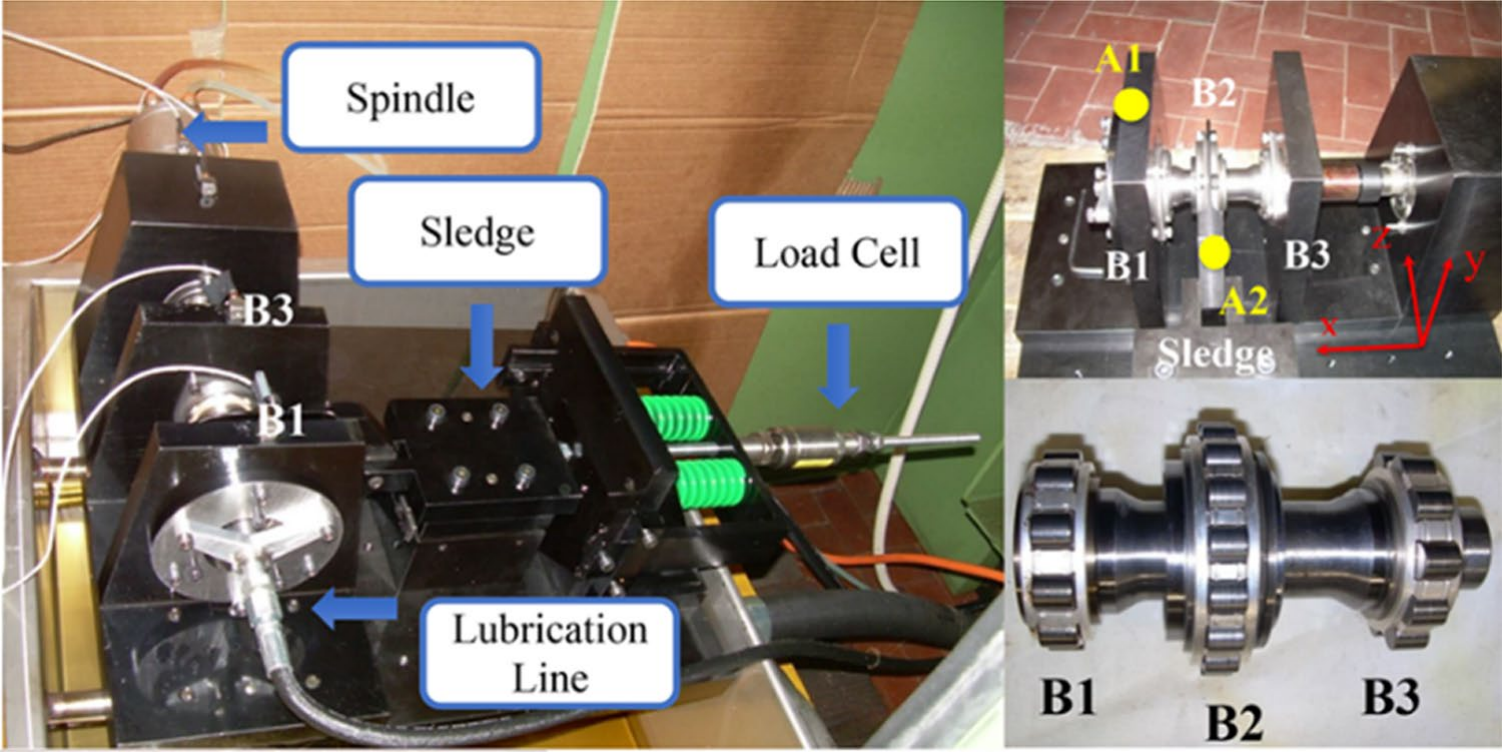
Turin Polytechnic University Bearing Laboratory.

The experimental setup for bearing testing and sensor configuration is illustrated in Fig. 11. In this study, Z-direction vibration data acquired from the sensor positioned at location A1 were utilized. The test rig primarily consists of a high-speed electric motor, the bearing unit under investigation, an axial loading mechanism, and an array of vibration sensors. The analyzed dataset encompasses three distinct bearing fault conditions, namely: (1) healthy state, (2) inner race defect, and (3) roller element defect. The data acquisition system was configured with a sampling frequency of 51.2 kHz, and for each operational condition, the sampling duration was maintained at 10 s.

The types of datasets used are as shown in Table 5:

**Table 5:** Fault diagnosis dataset specifications.

Data type	Label	Speed (rpm)
Normal	0	6000
Normal	1	12000
Normal	2	18000
Inner race defect	3	6000
Inner race defect	4	12000
Inner race defect	5	18000
Roller element defect	6	6000
Roller element defect	7	12000
Roller element defect	8	18000

#### Network accuracy testing

**Fig. 12:**
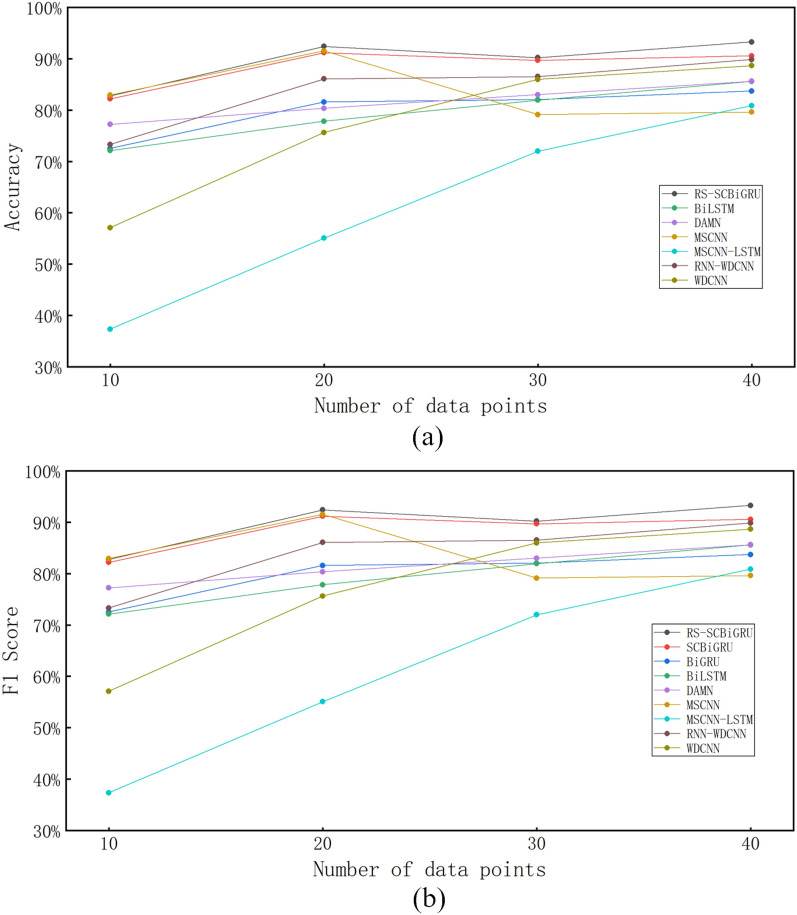
The relationship between different data volumes and accuracy.

To evaluate the fault diagnosis performance of the proposed model under varying quantities of training samples, comparative tests were conducted between the proposed method and other advanced algorithms. The experiments utilized four different scales of original data points, namely 10, 20, 30, and 40, for testing. The results were recorded in Table 6, and the changes in accuracy across different data volumes were illustrated in Fig. 12. These comparative analysis results fully demonstrate that the proposed method exhibits superior fault diagnosis capabilities across various data scales.

**Table 6:** Comparative performance evaluation of fault diagnosis methods across varying sample sizes.

Methods	10 samples		20 samples		30 samples		40 samples	
	Accuracy (%)	F1 score (%)	Accuracy (%)	F1 score (%)	Accuracy (%)	F1 score (%)	Accuracy (%)	F1 score (%)
RS-SCBiGRU	84.02	82.78	92.55	92.37	90.35	90.20	93.23	93.25
SCBiGRU	82.84	82.20	91.36	91.16	89.89	89.66	90.40	90.54
BiGRU	74.18	72.55	81.33	81.58	81.99	82.01	84.02	83.71
BiLSTM	73.88	72.13	78.05	77.82	82.21	82.94	85.96	85.59
DAMN	78.62	77.23	81.29	80.34	83.09	82.99	85.77	85.61
MSCNN	82.65	82.93	91.90	91.52	80.91	79.13	81.49	79.60
MSCNN-LSTM	38.32	37.36	56.90	55.09	73.95	72.00	82.40	80.86
RNN-WDCNN	74.51	73.30	86.28	86.07	86.82	86.52	90.16	89.85
WDCNN	58.75	57.12	76.85	75.62	86.30	85.96	88.72	88.63

As the amount of training data increases, the accuracy of algorithms generally improves, with the proposed method demonstrating the best overall diagnostic capability. For some algorithms, accuracy decreases when the data volume continues to increase, which is due to overfitting of the model under small-sample conditions, a point further corroborated by subsequent noise resistance tests.

#### Network noise resistance testing

**Fig. 13:**
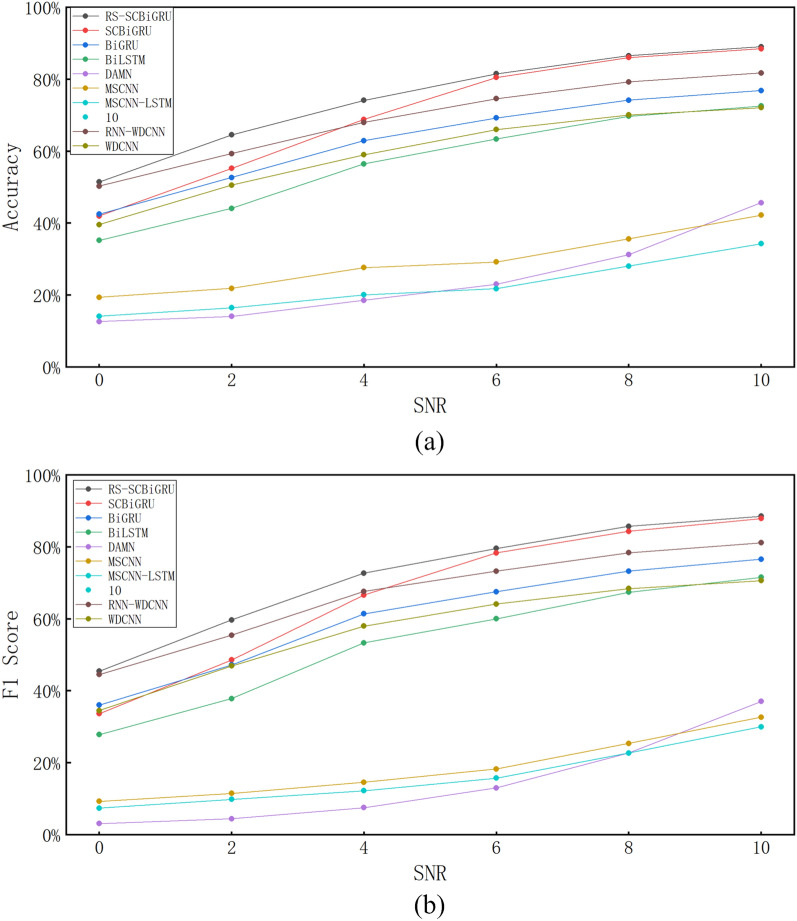
Network test results under different signal-to-noise ratios.

To evaluate the network’s anti-interference capability against bearing noise, noise was added to the bearing data, and relevant tests were conducted. Meanwhile, the proposed network was compared with several state-of-the-art fault diagnosis methods. Detailed test results are presented in Fig. 13 and Table 7.

**Table 7:** Performance comparison of different methods under varying signal-to-noise ratios.

Methods	0		2		4		6		8	
	Accuracy (%)	F1 (%)	Accuracy (%)	F1 (%)	Accuracy (%)	F1 (%)	Accuracy (%)	F1 (%)	Accuracy (%)	F1 (%)
RS-SCBiGRU	51.41	45.41	64.48	59.68	74.10	72.67	81.45	79.55	86.51	85.66
SCBiGRU	41.94	33.61	55.20	48.53	68.81	66.89	80.45	78.30	85.97	84.28
BiGRU	42.46	36.02	52.67	47.16	62.87	63.87	69.21	67.50	74.12	73.23
BiLSTM	35.16	27.78	44.08	37.79	56.41	53.30	63.37	59.97	69.67	67.34
DAMN	12.63	3.09	14.08	4.01	18.55	7.53	23.01	13.01	31.23	22.68
MSCNN	19.35	9.25	21.86	11.46	27.60	14.57	29.19	18.28	35.57	25.38
MSCNN-LSTM	14.13	7.38	16.48	9.84	20.04	12.24	21.78	15.70	28.02	22.67
RNN-WDCNN	50.25	44.48	59.28	55.40	67.97	67.57	74.56	73.23	79.21	78.32
WDCNN	39.55	34.50	50.53	46.90	58.96	57.96	65.99	64.08	70.01	68.36

Through comparative analysis, the following conclusions can be drawn: In small samples containing noise, the method proposed in this paper exhibits significant advantages in two crucial evaluation metrics: comprehensive fault diagnosis accuracy and network noise resistance capability.

#### Visual analysis

**Fig. 14:**
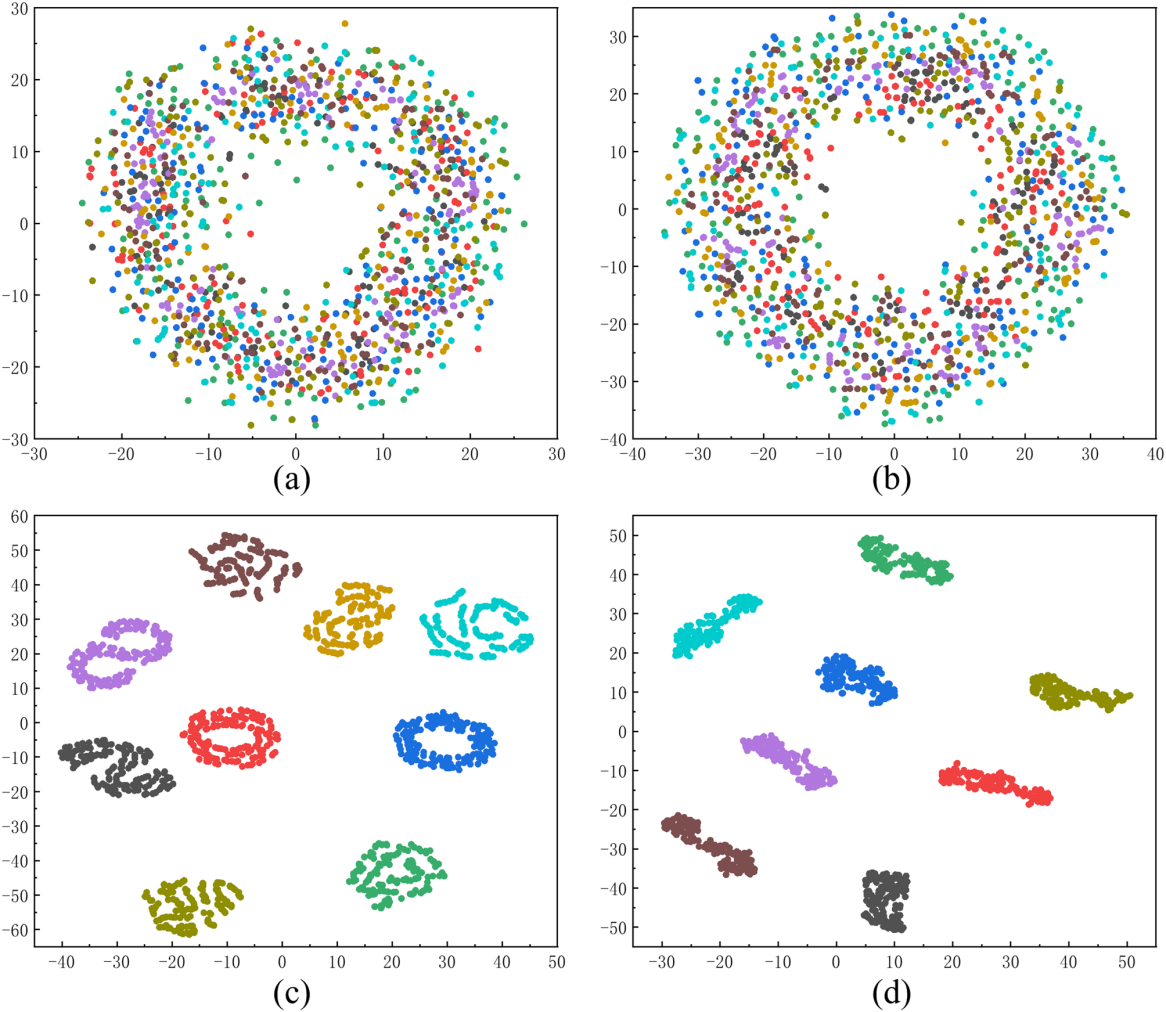
T-SNE fault characteristic map.

In Fig. 14a represents the initial feature state of fault data input. At this stage, different types of data exhibit a disordered and intermingled distribution pattern. Figure 14b shows the data distribution after processing with a large convolution kernel, where it can be observed that the data begins to display a certain degree of feature clustering, although it still appears quite chaotic overall. Figure 14c depicts the scenario after further processing by the convolutional layer, where the phenomenon of data clustering becomes more pronounced, but the degree of closeness between features has not yet reached an ideal state. Finally, at stage (Fig.14d), which is the final state of the network output, it is clearly visible that the features have formed distinct cluster structures, fully demonstrating the network’s excellent performance in processing such data.

## Conclusion

This paper proposes an RS-SCBiGRU model based on stochastic resampling and frequency-adaptive convolution for equipment health state recognition under partial information availability. By introducing stochastic resampling, the diversity of input data is enhanced, while the self-correcting convolution mechanism ensures effective utilization of fault characteristics. Experimental validation on the Jilin University bearing dataset (Case 1) and the Politecnico di Torino bearing dataset (Case 2) demonstrates that RS-SCBiGRU achieves superior diagnostic efficiency under noisy and variable operating conditions with partial data. The operational mechanism of the network is further elucidated through t-SNE visualization.

Although the network has made significant strides in fault diagnosis, there is still room for improvement in its diagnostic accuracy. Future research could consider integrating the RS-SCBiGRU framework with advanced technologies such as GANs and Siamese networks, with the aim of further enhancing the accuracy and efficiency of fault diagnosis. Moreover, the combination of the network proposed in this study with various advanced technologies holds the potential to address challenges such as zero-shot fault diagnosis and data imbalance, thereby ushering in innovative breakthroughs in the field of fault diagnosis.

## Data Availability

The data supporting this study are available in two forms: Public dataset: Available at ftp://ftp.polito.it/people/DIRG_BearingData via Politecnico di Torino repository. Restricted dataset: Available from the corresponding author upon reasonable request.
